# A new hybrid approach for MHC genotyping: high-throughput NGS and long read MinION nanopore sequencing, with application to the non-model vertebrate Alpine chamois (*Rupicapra rupicapra*)

**DOI:** 10.1038/s41437-018-0070-5

**Published:** 2018-03-24

**Authors:** S. Fuselli, R. P. Baptista, A. Panziera, A. Magi, S. Guglielmi, R. Tonin, A. Benazzo, L. G. Bauzer, C. J. Mazzoni, G. Bertorelle

**Affiliations:** 1 Department of Life Sciences and Biotechnology, University of Ferrara, Via L. Borsari 46, Ferrara, 44121 Italy; 2 Center for Tropical & Emerging Global Diseases, University of Georgia, 107 Paul D. Coverdell Center, 500 D. W. Brooks Drive, Athens, GA 30602-7394 USA; 3 Department of Biodiversity and Molecular Ecology, Research and Innovation Centre, Fondazione Edmund Mach, Via Edmund Mach 1, San Michele all’Adige, I-38010 Italy; 4 Department of Experimental and Clinical Medicine, University of Florence, Largo Brambilla, Florence, 3-50134 Italy; 5 Faculty of Science and Technology, Free University of Bozen-Bolzano, Piazza Università 5, Bolzano, Italy; 6 Laboratório de Fisiologia e Controle de Artrópodes Vetores, Instituto Oswaldo Cruz, Fiocruz, Rio de Janeiro, Brazil; 7 Berlin Center for Genomics in Biodiversity Research, Königin-Luise-Str. 6-8, Berlin, 14195 Germany

## Abstract

The major histocompatibility complex (MHC) acts as an interface between the immune system and infectious diseases. Accurate characterization and genotyping of the extremely variable MHC loci are challenging especially without a reference sequence. We designed a combination of long-range PCR, Illumina short-reads, and Oxford Nanopore MinION long-reads approaches to capture the genetic variation of the MHC II *DRB* locus in an Italian population of the Alpine chamois (*Rupicapra rupicapra*). We utilized long-range PCR to generate a 9 Kb fragment of the *DRB* locus. Amplicons from six different individuals were fragmented, tagged, and simultaneously sequenced with Illumina MiSeq. One of these amplicons was sequenced with the MinION device, which produced long reads covering the entire amplified fragment. A pipeline that combines short and long reads resolved several short tandem repeats and homopolymers and produced a de novo reference, which was then used to map and genotype the short reads from all individuals. The assembled *DRB* locus showed a high level of polymorphism and the presence of a recombination breakpoint. Our results suggest that an amplicon-based NGS approach coupled with single-molecule MinION nanopore sequencing can efficiently achieve both the assembly and the genotyping of complex genomic regions in multiple individuals in the absence of a reference sequence.

## Introduction

Loci of the major histocompatibility complex (MHC) encode receptors expressed on the surface of a variety of cells, where they play a central role in responses to protein antigens. MHC molecules bind and display peptides to T cells that can initiate the cascade of complex immune responses. MHC genes are among the most polymorphic genes in vertebrates, and their non-neutral genetic variation is directly linked to disease resistance, mate choice, and sexual attractiveness (Edwards and Hedrick 1998; Bernatchez and Landry 2003; Garrigan and Hedrick 2003). Their extreme genetic variation is concentrated in specific exons coding for the domain interacting with antigens (Abbas et al. 2015). There are two different types of MHC gene products, called class I and class II molecules. In this paper, we focus on the MHC class II *DRB* locus. In most vertebrates this gene has six exons, and exon 2, which is coding for the β-chain of the antigen-binding groove, is the most polymorphic (Hughes 2002). Efficient and reliable genotyping of MHC loci are not only hampered by the elevated number of polymorphic sites, but also by the presence of multiple copies of the same locus. For example, in several species, such as the bank vole, the rhesus macaque or the wild boar, the MHC II *DRB* can be a complex multilocus system (Doxiadis et al. 2000; Barbisan et al. 2009; Kloch et al. 2010).

The introduction of high-throughput next-generation sequencing methods (NGS) improved our ability to characterize MHC allelic diversity with reasonable time and costs in large samples, also reducing the risk of allelic dropout of less-preferentially amplified alleles (Babik 2010; Sommer et al. 2013). When more than a single locus is present, direct genotyping by Sanger sequencing is problematic and cloning may be necessary. Conversely, NGS approaches produce reads from single DNA template molecules and, at sufficiently high coverage, polymorphic positions with more than two alleles can be reliably called.

In a previous study, we characterized the Alpine chamois *DRB* locus using a PCR approach focused on exon 2 (Mona et al. 2008). The PCR products were directly Sanger sequenced. No more than two alleles were detected per single nucleotide polymorphism (SNP) and no more than two haplotypes were statistically assembled per individual, suggesting the presence of a single copy of the *DRB* locus (Mona et al. 2008). However, due to the stringency of our amplification, slight variations at primer binding sites for duplicated loci could have prevented their amplification. While exon 2 shows high-genetic variation, exons 1 and 3 are more conserved within and between species, and, putatively, across multiple loci of the same individual. One possibility to decrease the stringency of our assay is therefore to avoid the variable exon 2 for primers annealing and design primers on exons 1 and 3. Besides increasing the probability to co-amplify multiple loci, if present, this approach extends the characterization of the *DRB* genomic region in our species. More in detail, we designed primers on *DRB* exons 1 and 3 and set-up a long-range PCR coupled with Illumina NGS sequencing to a very high coverage. Long-range PCR is an efficient, robust, and cost-effective approach for sequencing candidate genomic regions with NGS platforms (Jia et al. 2014; Deiner et al. 2017). The resulting amplicon (defined as a set of sequences derived from the same PCR that can include allelic copies from the same or multiple loci) captures the most variable region of the *DRB* locus, i.e., intron 1- exon 2- intron 2, spanning about 16 Kb in *B. taurus* (Ensembl: ENSBTAG00000013919) and 9 Kb in *Ovis aries* (GenBank AM884914). To assemble the Illumina short reads into a scaffold we generated single long reads capable of covering the entire amplicon with the portable MinION sequencer developed by Oxford Nanopore Technologies (Clarke et al. 2009; Ward and Kim 2015). Our sequencing project was part of the MinION Access Programme (MAP) launched by Oxford Nanopore. MinION sequencing is rather inexpensive and faster than most established DNA sequencing methods, and the throughput is particularly suitable for amplicon experiments (each consumable flow cell is expected to generate 5–10 Gb of DNA sequence data, nanoporetech.com/products/minion). We estimated the error rate of our MinION experiment and we polished the long-reads consensus exploiting the lower error rate of short reads produced by Illumina. Using this hybrid strategy, we established a reference sequence of most of the *DRB* locus in a non-model species. This allowed us to characterize the genotype of six genetically heterogeneous individuals at SNPs, numerous indels, and one intronic short tandem repeat (STR). Based on these results we could statistically infer the haplotype phase and detect the presence of a recombination breakpoint. The characterization of this region could be now easily extended to additional chamois individuals.

## Methods

### Samples

Chamois tissue samples (skeletal muscle) were collected between 2010 and 2013 from wild animals in the Dolomiti Bellunesi National Park (eastern Italian Alps). Tissues were preserved in ethanol until DNA extraction.

### DNA extraction and Sanger sequencing of MHC class II DRB exon 2

**Fig. 1:**
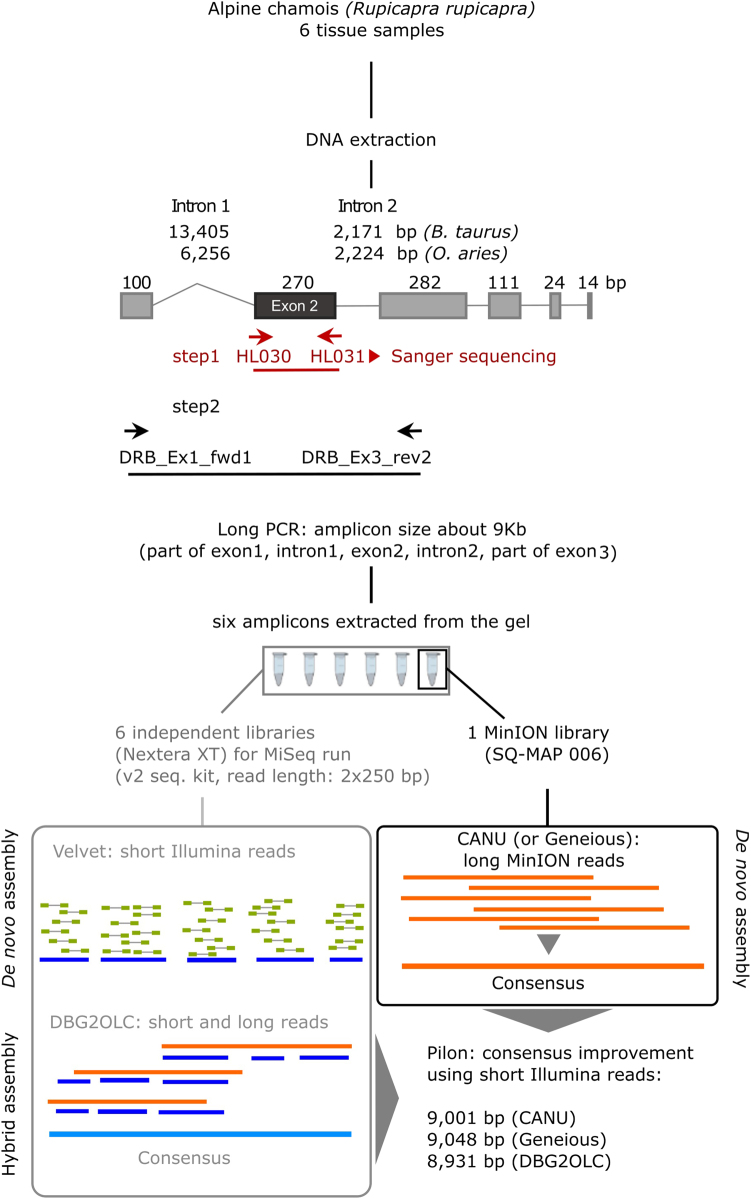
Laboratory procedure used to obtain MHC II *DRB* short and long amplicons, and assembly pipelines. The short amplicon was sequenced by standard Sanger sequencing (step 1), while the long amplicon was sequenced with Illumina MiSeq and nanopore MinION after gel extraction (step 2). The gene structure is based on *Bos taurus* (Ensembl: ENSBTAG00000013919) and *Ovis aries* (GenBank AM884914) structures. Boxes: coding regions, lines: introns, horizontal arrows: PCR primers

Genomic DNA was extracted from alcohol preserved (95% ethanol) tissues using the DNeasy Blood & Tissue Kit (QIAGEN). Amplification of a 302 bp *DRB* exon 2 fragment was achieved using primers HL030: 5′-ATCCTCTCTCTGCAGCACATTTCC-3′ and HL031 (5′-TTTAAATTCGCGCTCACCTCGCCGCT-3′) complementary to the boundary between intron 1-exon 2 and exon 2-intron 2, respectively (Fig. 1) (Schaschl et al. 2004). PCR amplification was performed as in Barbisan et al. (2009). The 302 bp fragment was Sanger sequenced using PCR primers.

### Long-range PCR: primer design and reaction conditions

With the aim of capturing *DRB* copies that may have passed undetected in our previous study (Mona et al. 2008), we designed primers on *DRB* conserved regions, putatively shared by multiple copies of the locus (Fig. 1). Exons 1, 2, and 3 nucleotide sequences were compared within and between species to estimate their level of conservation. The proportion of variable nucleotide positions per exon within *B. taurus DRB* calculated from the Ensembl database (www.ensembl.org) is 0.06 for exon 1 and 0.11 for exon 3, while it increases to 0.21 for exon 2. Between Bovidae species (Table S1a, median time of divergence between chamois and cattle: 24.60 MYA, interval 21.41–29.46 MYA, www.timetree.org) the proportion of variable sites becomes 0.19, 0.26, and 0.09 for exons 1, 2, and 3, respectively. Given the higher proportion of conserved sites, primers were designed on exons 1 and 3. This approach required to set-up a long-range PCR reaction for a range of length between 9 Kb (based on *O. aries* GenBank AM884914) and 16 Kb (based on *B. taurus* Ensembl: ENSBTAG00000013919). The intron length of chamois’ *DRB* was unknown, since only the cDNA had been deposited in GenBank (AF336340.1). The inclusion of intron 1, exon 2, and intron 2 was expected to capture the most variable part of the locus.

Long-PCR primers located in exons 1 and 3 were designed using Primer3 (biotools.umassmed.edu/bioapps/primer3_www.cgi) and considering (1) *B. taurus DRB* polymorphic sites available in Ensembl database, (2) conserved regions in the alignment between orthologous *DRB* loci from 15 different Bovidae species (Table S1a), and (3) *DRB*-specific regions resulting from the alignment with the paralogue *DQB* from *B. taurus* and *O. aries*, showing a high degree of sequence similarity (Table S1b, Supporting information for accession numbers). The primer pair DRB_Ex1_Fwd1 5′-GATGCTGAGCCCTCCCCTGG-3′ and DRB_Ex3_Rev2 5′-CCGGAACCACCTGACTTCAA-3′ was used for the *DRB* long-range PCR (Fig. 1).

Long-range PCR conditions were as follows: the 50-µl reaction mixture contained 1 U of Platinum Taq DNA Polymerase High Fidelity (Life Technologies), 1X buffer HiFi, 2 mM Mg(SO)_4_, 0.2 mM each dNTP, 0.2 µM each primer, and 50–100 ng of genomic DNA. The thermal cycling profile included an initial denaturation at 94 °C for 1 min followed by 35 cycles of 94 °C for 20 s, 62 °C for 30 s, 68 °C for 15 min. The PCR product was run on 1% low melting SeaPlaque™ GTG™ Agarose gel; the expected ~9 Kb band was cut and extracted using the MinElute Gel Extraction Kit (QIAGEN). Both long-PCR products and fragments extracted from the gel were run on a Bioanalyzer 2100 (Agilent) using the DNA 12000 chip assay.

### MiSeq sequencing

The library preparation was done according to the NexteraXT protocol (Illumina) for each sample separately. Briefly, 1 ng of each PCR product was subjected to tagmentation with 5 µl of Amplicon Tagment Mix, a step that enzymatically cleaved the DNA to an expected fragment size of approximately 300 bp. Illumina adapters and barcodes were introduced during the indexing step via PCR. The samples were quantified using Qubit dsDNA HS Assay kit (Thermo Fisher Scientific) and Agilent Bioanalyzer, and subsequently pooled equimolary. The pool was sequenced on an Illumina MiSeq (Illumina) run using a v2 nano sequencing kit (2 × 250 bp).

### MinION sequencing

After gel purification, one of the six amplicons was sequenced in parallel with MinION system. Specifically, the amplicon was selected to be from a homozygous Ruru-DRB*01 individual (GenBank: AY368437.1) based on exon 2-Sanger sequencing results (PDB70, Table 1). After quantification with Qubit dsDNA HS Assay kit, 1 µg of amplicon was end-repaired and dA-tailed using NEBNext modules (New England BioLabs) together with 5 µl of lambda phage DNA (control DNA, CS, ONT). After these steps, specific adapters (the leader adapter bound to a motor protein and the hairpin adapter) were ligated to the end repaired-dA tailed amplicon using Genomic DNA Sequencing Kit MAP-006 (Oxford Nanopore). Finally, HP tether with affinity for the polymer membrane was added to the mix. Enrichment for molecules containing bound motor protein was performed using MyOne C1 Streptavidin magnetic beads (Life Technologies). The DNA library, or pre-sequencing mix, was eluted in 25 µl of elution buffer. The flow cell (R7.3 chemistry) was then conditioned by loading 500 µl of the priming mix (fuel mix, running buffer, and nuclease free water), waiting 10 min, and repeating the two steps. The pre-sequencing mix was added to a solution of fuel mix, running buffer and nuclease free water, then 150 µl of the final solution were loaded into the flow cell. The sequencer was run for 48 h, and reloaded after 24 h, just before the second “mux scan.”

**Table 1:** Sanger and Illumina sequencing in six different chamois

Code	Exon 2 genotype	Filtered paired-end Illumina reads	Coverage
PDB44b	*1/*19	81,284	×3910
PDB47	*1/*19	147,388	×6294
PDB60b	*1/*1	78,425	×2154
PDB61	*19/*19	102,110	×4341
PDB66	*19/*19	67,599	×2797
PDB70^a^	*1/*1	140,721	×6220

^a^Sample analyzed with MinION

### Bioinformatics and statistical analysis

#### MiSeq data analysis

The Illumina reads of the six different amplicons were filtered using Trimmomatic v0.36 (Bolger et al. 2014) following specific criteria. We performed the trimming of the Illumina adapters and of the 3′ and 5′ ends (leading and tailing minimum base quality = 5), we set-up a minimum read length size of 40 bp, and to ensure a good average quality of the trimmed reads, we set-up an average quality threshold of 30 in a sliding window of four bases. After filtering, we performed a de novo assembly using the Illumina reads from one individual’s amplicon, PDB70, the same that was sequenced with MinION nanopore. De novo assemblers are not optimized for extremely high depth of coverage: de novo assembly is computationally intensive and redundancy reduces the performance without increasing the power to resolve gaps due to repetitive sequence traits where coverage drops to very low values (Zerbino 2010). Therefore, we randomly selected three subsets of the total reads to create three respective fastq files with the tool seqtk (https://github.com/lh3/seqtk), the size parameter setting used for each subset was 100,000 reads. A de novo assembly of each of the three subsets having a coverage of about ×100 was performed with Velvet (best k-mer size: 21, selected by Velvet optimizer) (Zerbino and Birney 2008).

#### MinION data analysis and reads alignment

The base calling of the reads produced by the MinION sequencer was performed via the Metrichor agent (provided by Oxford Nanopore Technologies) and the two-dimensional (2D) nanopore reads, which passed the quality filter were converted from *.fast5* to *.fastq* using Poretools (Loman and Quinlan 2014). Reads shorter than 1 kb were excluded from the analysis to avoid problems during the assembly process in presence of challenging regions, such as homopolymers or repeat motifs. Reads longer than 9 Kb were individually analyzed to determine whether we could detect significant similarities between those reads and the amplified locus. Specifically, the reads were blasted against the NCBI nucleotide collection (nr/nt) and genomic survey sequences (gss), using the programs megablast (for highly similar sequences), discontinuous megablast (more dissimilar sequences), and blastn (somewhat similar sequences). The nature of the reads that showed partial significant similarity to the *DRB* locus was further investigated to consider the possibility that a second MHC locus had been amplified and sequenced, even if less efficiently. To identify artefacts or sequence chimeras, we used the Proovread 2.12 workflow (Hackl et al. 2014). First, short Illumina reads are mapped onto putatively erroneous and chimeric nanopore long reads. Then, during the consensus generation the majority of errors are removed and possible chimeric break points are identified. Finally, the resulting reads are trimmed using a quality cutoff and chimera annotations to generate high-accuracy long reads.

Three different approaches were used to avoid bias in the final assembly due to assembly method. First, we performed a long-read-only de novo assembly of nanopore reads using CANU version 1.4 (derived from Celera assembler) (Koren et al. 2017) and the whole pipeline of correcting, trimming, and assemble nanopore-raw reads. The all default three stages were made using the “-nanopore-raw” parameter that is recommended for non-corrected nanopore read datasets and also “genome size =9 k,” estimated size obtained by our amplified genomic region. Second, we performed a hybrid approach based on the contig assembly by short reads (in our case the Velvet assembly described above) and the scaffolding by long reads using DBG2OLC (Ye et al. 2016) using the “k 17 AdaptiveTh 0.001 KmerCovTh 2 MinOverlap 5 RemoveChimera 1” parameters, as suggested for long-read coverages <~×50, to select which regions will be considered as good quality for the anchor between the Illumina contigs and the our long-read dataset.

A third set of analyses was performed using the software Geneious v8.1.8 (Kearse et al. 2012). A random subset of 20 reads about 9 Kb long (i.e., probably covering the whole amplicon) was multi-aligned to create a first nanopore-based consensus. All the reads ≥1000 bp were aligned to this first scaffold to produce the final consensus sequence. The same procedure was repeated starting from a second set of 20 randomly chosen 9 Kb reads. All the alignments were performed using the Geneious aligner with intermediate parameters between high and medium sensitivity: five iterations; maximum mismatches per read 40%; maximum gap per read 20%; maximum gap size 50 bp.

The results of the three methods were submitted to Pilon version 1.21 (Walker et al. 2014) that uses the Illumina read data to polish all base calls and improve the quality of the assembly. The parameters used for this polishing stage included “-diploid“ so heterozygous SNPs would not be called as errors, and the mapping quality used in the BWA-mem alignment. The identity between each of the three final assembly and *O. aries DRB* (GenBank AM884914, Table 2) was obtained by the program nucmer (NUCleotide MUMmer, Kurtz et al. 2004).

**Table 2:** Assembly pipelines and descriptive statistics of PDB70 amplicon reads obtained with Illumina and MinION nanopore

Pipeline	Type	Input	Error corrector	Contig assembler	Consensus size (bp)	GC%	Coverage^a^	Identity between chamois and *Ovis aries* DRB (GenBank AM884914)
CANU	De novo	MinION	Pilon	CANU	9001	40.86	×7139.92	95.81%
Geneious	De novo	MinION	Pilon	Geneious algorithm	9048	40.86	×7120.34	95.81%
DBG2OLC	Hybrid	Illumina, MinION	Pilon	Velvet (short reads)	8931	40.87	×7065.76	91.75%

^a^The coverage was calculated using Samtools (Li et al. 2009) considering Illumina reads mapped to Pilon-polished CANU, Geneious and DBG2OLC assemblies, respectively

#### Estimation of the nanopore MinION error rate

The 2D reads of lambda phage spike-in DNA (control DNA, CS, ONT) were aligned against the lambda reference genome by using three different mapping tools: BWA (Li and Durbin 2009), BLASR (Chaisson and Tesler 2012), and GraphMap (Sović et al. 2016). For each tool, parameters were chosen on the base of the recommendations and tweaking made by other MAP and MinION Analysis and Reference Consortium participants and reported in Jain et al. (2015). BWA version 0.7.12 was used with the “-x ont2d” that was properly devised for the alignment of ONT 2D reads. BLASR (bix.ucsd.edu/projects/blasr/) was applied to nanopore reads with the parameters -sdpTupleSize 8 -bestn 1 -m 0 (Jain et al. 2015). GraphMap was applied to nanopore reads with the Gotoh alignment with anchored approach (“anchorgotoh” option). Results were obtained by parsing BAM files with SAMtools (Li et al. 2009) and in-house bash and R scripts (Supporting information_error rate estimation).

#### Polymorphism data analysis

The filtered Illumina reads of the six *DRB* libraries were aligned to the final consensus using BWA-mem Version 0.7.12 (Li 2013), the “-M” parameter was used to mark shorter split hits as secondary, to make the output compatible for SNP call step. Removal of PCR duplicates and local realignment around indels were performed using Picard (http://broadinstitute.github.io/picard/) and GATK ((McKenna et al. 2010). Next, polymorphic positions and genotypes of each of the six amplicons were called with the GATK module HaplotypeCaller.

The datasets generated and analyzed during the current study are available in the NCBI Sequence Read Archive (SRA, accession number SRP124753).

### Haplotype reconstruction and detection of recombination signals

Haplotypes were statistically inferred using PHASE v2.1.1 (Stephens et al. 2001). The program was run three times with different random seeds (10^5^ iterations per run with 10% burn-in period). In the analysis, we included 109 polymorphic positions of which 102 were SNPs and seven were indels, while STRs and homopolymers were not considered. To detect recombination within the amplicon we employed four different algorithms: RDP (Martin and Rybicki 2000), GENECONV (Padidam et al. 1999), Chimaera (Posada and Crandall 2001), and MaxChi (Maynard Smith 1992), available in the package RDP4 (Martin et al. 2010), with default settings for linear sequences.

## Results

### Long-range PCR

An aliquot of the long-range PCR product was run on the Bioanalyzer DNA 12000 chip assay, which showed the presence of five fragments of different length (~480, 930, 2000, 2900 bp, and 8.8 Kb, Fig. 1a supporting information). The following analysis showed that the larger fragment corresponds to the *DRB* amplicon. After gel extraction, the three smaller fragments were no longer present, but a smear from 6 to 11 Kb was detected around the 8.8 Kb peak (Fig. 1b supporting information).

### De novo assembly of MiSeq Illumina reads

The Illumina sequencing of all six libraries produced 1,243,596 paired-end reads. After filtering, 617,527 reads were retained, 140,721 of which could be attributed to PDB70 (Table 1). A de novo assembly of PDB70 Illumina reads produced 14 non-overlapping contigs, from 788 to 3451 bp long, with mean and median length equal to 1555 and 1205 bp, respectively. To identify *DRB*-specific sequences, we blasted the 14 contigs against the NCBI Nucleotide collection and Genome survey sequences. Nine contigs mapped to non-MHC regions of *Ovis canadiensis* genome (CP011896.1), suggesting that part of chamois genome has been amplified during the long-range PCR due to the non-specific binding of the primers. Five contigs mapped to the *DRB* locus of *Ovis* species, including *O. aries* (GenBank ID: AM884914), and one of them carried exon 2 (length of the contig: 1060 bp). These five contigs cover in total 6628 bp, indicating that the de novo assembly failed to reconstruct about 2000 bp of the original amplicon. The alignment to the *O. aries* DRB sequence showed that the beginning of the amplicon and four regions between the contigs remained uncharacterized (Fig. 1). In two cases the lack of contigs corresponded to the presence in *Ovis* of low-complexity regions, specifically a stretch of 13 T at the end of the first contig, and STRs located just downstream of exon 2.

### Assembly of MinION nanopore reads

**Fig. 2:**
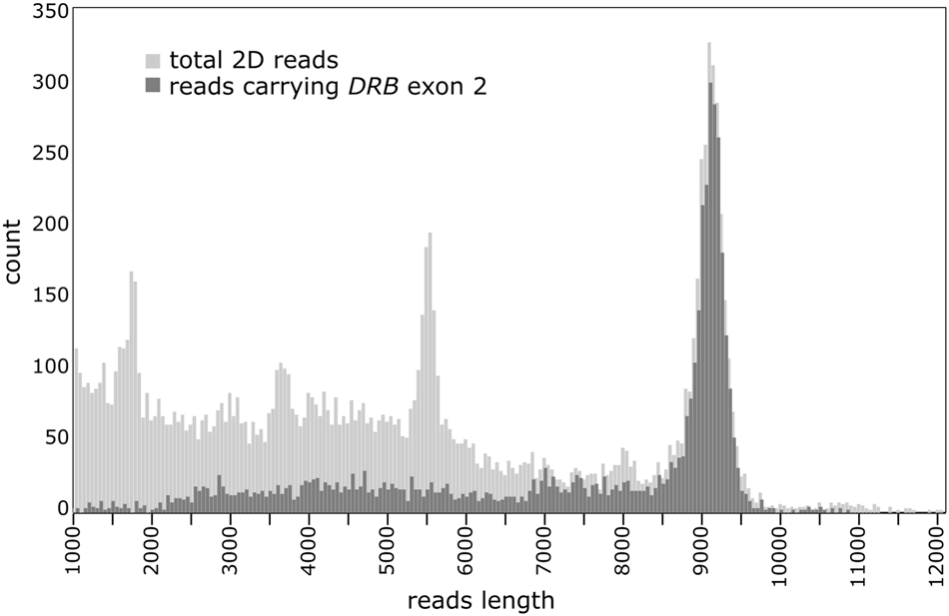
Distribution of 2D reads between 1 and 12 Kb length produced by the MinION run

The MinION run produced 40,585 reads corresponding to 64.8 Mb yield, lower than the expected throughput, but similar to what has been obtained in other experiments with the same version of the MinION technology (Ip et al. 2015). Approximately one third of the reads (14,520) were in 2D, with an average, median and mode length of 4186, 3575, 3656 bp, respectively. The distribution of the reads of different length is shown in Fig. 2.

We excluded from the analysis 2480 reads shorter than 1 Kb and 113 reads longer than 10 Kb, the latter corresponding to reads exceeding the length of the amplicon. Of these extra-long reads 24 show very short regions (<300 bp) with significant similarity in the NCBI nucleotide collection and gss databases. The remaining 89 extra-long reads showed partial significant similarity to *O. aries DRB* locus (from a maximum of 9742 bp to a minimum of 30 bp, mean 1912, and median 1354), and non-*DRB* sequence either mapping on several different regions of *O. canadiensis* genome (CP011896.1), or without any significant similarity in the searched databases. Our chimera detection analysis identified chimeric break points in four of the 113 extra-long reads and trimmed 105 low-quality positions, producing a new set of 208 higher quality sequences (range length: 515–10,122 bp). The coverage of a further short reads alignment to the new set of sequences was still very low and unevenly distributed, suggesting that the reads exceeding the amplicon length are artefacts comprising part or the entire *DRB*-specific amplicon and/or other non-specific long-range PCR products.

The long-read-only assembly by CANU spanned 8806 nucleotides. After polishing the assembly with Pilon, 126 positions (123 deletions and three substitutions) were corrected, and we obtained a 9001 bp consensus sequence (Table 2 and Fig. 1) and a coverage of ×7139.92. The Geneious approach led to a consensus sequence of 9048 bp very similar to that obtained by CANU, with the exception of few nucleotide differences in three homopolymeric regions (position 1883, 2055, 7954 of the CANU assembly) and in the STR downstream of exon 2. The DBG2OLC approach produced a shorter and more distant consensus (8931 bp). The comparison to *O. aries DRB* reflects the similarity among the three consensus sequences, with CANU and Geneious assemblies showing a higher identity to *Ovis* sequence than DBG2OLC one (Table 2). These results suggest that the locus region characterized in our study is highly conserved between the two species. The CANU assembly was used as a reference sequence in further analyses.

### Polymorphism data analysis, haplotype reconstruction, and detection of recombination signals

**Fig. 3:**
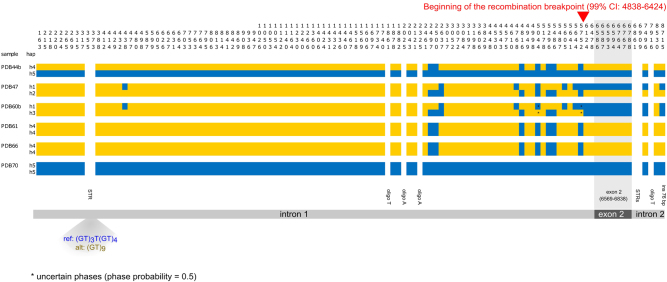
Graphical representation of individual inferred haplotypes. Vertical numbers represent the position on the MHC amplicon. Different colors indicate reference allele (blue) and alternative allele (yellow). In white variable positions of undefined genotype. The shaded triangle shows the two alleles of the STR identified in intron 1

The distribution of polymorphic positions across the six individuals of known exon 2 genotype (hereafter *1/*1, *1/*19, and *19/*19, Table 1) is shown in Fig. 3. With some exceptions (one polyT and two polyAs in intron 1, the STR-rich region just downstream of exon 2, and one polyT in intron 2) genotypes could be defined for all individuals for both substitutions (*n* = 102) and indels (*n* = 7). The polymorphic dinucleotide STR located in intron 1 (position 369 of the reference sequence) showed two alleles in our samples, the reference (GT)_3_T(GT)_4_ and the alternative allele (GT)_9_ whose genotypes are indicated in Fig. 3. Our analyses confirmed the previous Sanger sequencing of exon 2. By applying the statistical approach implemented in the software PHASE we could infer the presence of five different haplotypes in our samples (Fig. 3). Despite this low number, three of the four algorithms used for detecting recombination (i.e., GENECONV, Chimaera, and MaxChi) inferred the presence of a breakpoint within the amplicon sequence (*p*-values <10^−3^). In particular, the breakpoint was estimated to begin at position 5777 of the consensus sequence (99% CI 4838–6424), whereas the end of the breakpoint could not be determined. Since exon 2 goes from nucleotide 6569 to nucleotide 6838, the results suggest the presence of two blocks of association, the first spanning intron 1 and the second spanning exon 2 and intron 2 (Fig. 3). More haplotypes should be analyzed to confirm this preliminary result.

### Estimation of the nanopore MinION error rate

**Fig. 4:**
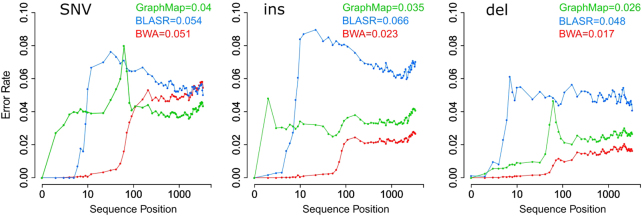
MinION sequencing error rate estimation using three mapping algorithms. The error rate for single nucleotide variants (SNV, average error: 4.8%), insertions (INS, average error: 4.1%), and deletions (DEL, average error: 3.0%) was estimated as a function of sequence position

In order to obtain a raw estimation of nanopore data error rate for the three main sources of local errors (mismatch, deletions, and insertions) we aligned sequences against the lambda phage reference genome by using three different mapping algorithms. In particular, all the sequences were aligned by using three state-of-the-art mapping tools (BWA (Li and Durbin 2009), BLASR (Chaisson and Tesler 2012), and GraphMap (Sović et al. 2016, see Methods), and we then calculated the number of bases that are substituted, inserted, and deleted with respect to the reference genome as a function of sequence position (Fig. 4).

Although the results of these analyses give a combination of sequencing and alignment errors, the use of three different mapping strategies allowed us to mitigate the alignment effect obtaining a good estimation of sequencing errors.

The three alignment algorithms return different results in handling the three error categories, with GraphMap that obtains the lower rate in substitution errors and BWA in insertions and deletions (Fig. 4). Figure 4 shows that the global error rate of nanopore reads is 9–11%, in accordance with the results previously reported in other papers (Magi et al. 2016).

## Discussion

In this paper, we propose and test a method to genotype the MHC class II *DRB* locus in a non-model species where the genome is not available, the Alpine chamois, using a hybrid approach based on Illumina short reads and nanopore long reads sequencing. This approach combines the low error rate of the Illumina platform with the ability of nanopore long reads to assemble complex genomic regions. It also increases the chances to detect duplicated genes.

The presence of multiple copies sequenced at the same time can leave signatures in the alignment. For example, tri-allelic SNPs may be detected, the frequency of the two alternative nucleotide in bi-allelic SNPs may be uneven (25:75% instead of 50:50%), and an elevated level of heterozygosity may characterize the whole sequence. None of these signatures were detected in our alignments, despite the extreme depth of coverage (Table 1). However, we cannot exclude that multiple copies are present only in some unsampled individuals of the population as observed in other species (Barbisan et al. 2009). Alternatively, long-range PCR primers may be specific for just one *DRB* copy. This second explanation seems less plausible since exons 1 and 3 coding for the structural part of the MHC are conserved, as shown by within and between species genetic variation estimations, unless the locus has become a pseudogene. Additionally, we observed the co-amplification of short amplicons resulting from non-specific binding of primers. This suggests that our amplification cycle is rather permissive (68 °C of elongation for 15 min and 35 cycles) and eventual duplicated loci with some mismatches in exons 1 and 3 annealing sequences would have been amplified as well.

Notwithstanding the high coverage of the Illumina run, the de novo assembly of the short reads produced 14 independent contigs, and no scaffold could be assembled. We could infer the order of the contigs by their alignment to the *O. aries* sequence, but still several regions remained uncharacterized. Sanger sequencing with primer walking would not be feasible given the presence of several heterozygous indels and long stretches of STRs within the amplicon (Fig. 3). In this situation, the long reads produced by a single run of the MinION nanopore sequencer allowed us to build a consensus of the entire amplicon. Importantly, by aligning the Illumina reads to this nanopore consensus, we corrected the long-reads errors and we obtained the genotype of each of the six sampled individuals (Fig. 3). In the amplicon’s regions carrying homopolymers the genotype call was not reliable. As recently observed by sequencing the *Caenorhabditis elegans* genome with both Illumina and MinION nanopore approaches (Tyson et al. 2017), long reads are necessary to elucidate the structure of complex repetitive regions, but homopolymeric tracts are problematic. Considering the error rate expected in the long reads of our experiment, we sequenced a single homozygous individual with MinION to obtain the least possible number of problematic regions and efficiently guide the assembly of the Illumina reads. Recent improvements in the chemistry of the MinION have overcome the majority of issues associated with low yield and high-error rates (Jain et al. 2017). Running multiple barcoded MHC amplicons with the improved MinION version could provide the phases of additional haplotypes, and possibly improve the guidance role of the long reads. We note however that, even in its present MinION version and with a small sample size, the proposed method allowed the statistical inference of the haplotypes carried by each individual (Fig. 3). Also, by extending our typing beyond the coding exon 2, we could detect the presence of a recombination breakpoint at the end of intron 1, although more haplotypes should be analyzed to draw robust conclusions on the locus rearrangement. In summary, our workflow proved to be efficient for genotyping, phasing, and detecting signature of recombination at one of the most heterogeneous genomic regions of vertebrates. Additionally, the inclusion of the intronic regions in the amplicon allowed the identification of one polymorphic STR in intron 1 whose variation could be determined directly from the alignment, and a couple of variable STRs in intron 2 for which a PCR assay could easily be developed.

In conclusion, we suggest that our approach that integrates few nanopore runs with Illumina sequencing of barcoded individuals can extend and improve our ability to understand the structure and the level of MHC variation in non-model species. Following the same protocol, more amplicons could be genotyped in a single Illumina run to characterize entire population samples. Specifically, our MiSeq run produced an extremely high depth of coverage for each amplicon, but we show how a few random subsets of coverage ×100 each are sufficient for the assembly of the consensus sequence. Even conservatively considering a twice as large coverage, a single MiSeq run using Kit v2 Nano with an output of 500 Mb would allow the simultaneous typing of 250 barcoded individuals, and the variants calling in species whit rare or no evidence of multilocus amplification. For more complicated systems with multiple co-amplifying copies a higher coverage may be required and the number of samples that can be multiplexed should be specifically calculated (Galan et al. 2010; Biedrzycka et al. 2017). By genotyping several haplotypes with our approach we could obtain detailed information to reconstruct the evolutionary relationships between different alleles beyond the 270 nucleotides of exon 2. In particular, as suggested by our results, typing intronic variation increases the power to infer intragenic recombination, which is a mechanism known to play an extremely important role in the evolution of the MHC loci (Andersson and Mikko 1995). The extension of our approach to other individuals carrying different haplotypes will allow the investigation of further evolutionary aspects, such as the age of different alleles and their fine geographic distribution.

A better characterization of this highly variable genetic region could have a positive impact in conservation genetics studies (Sommer 2005; Zhu et al. 2013; Rico et al. 2016), and in general for the understanding of the genetic determinants of pathogen resistance (Hedrick 2002; Froeschke and Sommer 2005; Worley et al. 2010) and the patterns of host–parasite coevolution (Piertney and Oliver 2005; Spurgin and Richardson 2010; Eizaguirre et al. 2012).

### Data archiving

Sequence data have been submitted to SRA accession number SRP124753 (https://www.ncbi.nlm.nih.gov/sra/?term=SRP124753)

## Electronic supplementary material

Supplementary Information error rate estimation scripts(TXT 11 kb)

Supplementary Information ReadMe error rate estimation(TXT 0 kb)

Supporting Information Table S1(DOCX 12 kb)

Supporting Information Figure S1(PDF 367 kb)
