# Supplementary material for: A new hybrid approach for MHC genotyping: high-throughput NGS and long read MinION nanopore sequencing, with application to the non-model vertebrate Alpine chamois (Rupicapra rupicapra)
Source: Heredity (Edinb). 2018 Mar 24;121(4):293–303. doi: 10.1038/s41437-018-0070-5 (PMC6133961; doi:10.1038/s41437-018-0070-5)
Supplement: Supplementary file 3 — Supporting Information Table S1(DOCX 12 kb) [file 41437_2018_70_MOESM3_ESM.docx]

Supporting information

Table S1: accession numbers of MHC class II orthologues and paralogues

a: DRB orthologues in *Caprinae* (from GenBank) and *Bos taurus* (from Ensembl)

Bos_taurus_ENSBTAT00000018484 cds

Rupicapra_rupicapra_gi|18033488|gb|AF336340.1|

Ovis_aries_gi|165999|gb|M73984.1|

Ovibos_moschatus_gi|14625488|gb|AF387317.2|

Ovis_canadensis_gi|386877021|gb|JN081876.1|

Ovis_canadensis_gi|386877019|gb|JN081875.1|

Ovis_canadensis_gi|386877017|gb|JN081874.1|

Ovis_canadensis_gi|386877015|gb|JN081873.1|

Ovis_canadensis_gi|386877013|gb|JN081872.1|

Ovis_canadensis_gi|386877011|gb|JN081871.1|

Ovis_canadensis_gi|386877009|gb|JN081870.1|

Ovis_canadensis_gi|386877007|gb|JN081869.1|

Capra_hircus_gi|2575822|dbj|AB008346.1|

Capra_hircus_gi|2575820|dbj|AB008345.1|

Hemitragus_jemlahicus_gi|18033490|gb|AF336341.1|

b: DRB paralogues (DQB) in *Bos taurus* (from Ensemble) and *Ovis aries* (from GenBank)

*Bos taurus* ENSBTAG00000019588

ENSBTAG00000021077

*Ovis aries* EU176819.1 (DQB1 and DQB2)
