# Supplementary material for: A new hybrid approach for MHC genotyping: high-throughput NGS and long read MinION nanopore sequencing, with application to the non-model vertebrate Alpine chamois (Rupicapra rupicapra)
Source: Heredity (Edinb). 2018 Mar 24;121(4):293–303. doi: 10.1038/s41437-018-0070-5 (PMC6133961; doi:10.1038/s41437-018-0070-5)

Supporting information. Figure S1
Results of two runs of Bioanalyzer DNA 12000 chip assay

Figure S1a

PDB70 amplicon obtained by long-range PCR with primers DRB_EX1_fwd1 and DRB_EX3_rev2 (see step2 in Figure 1, main text)

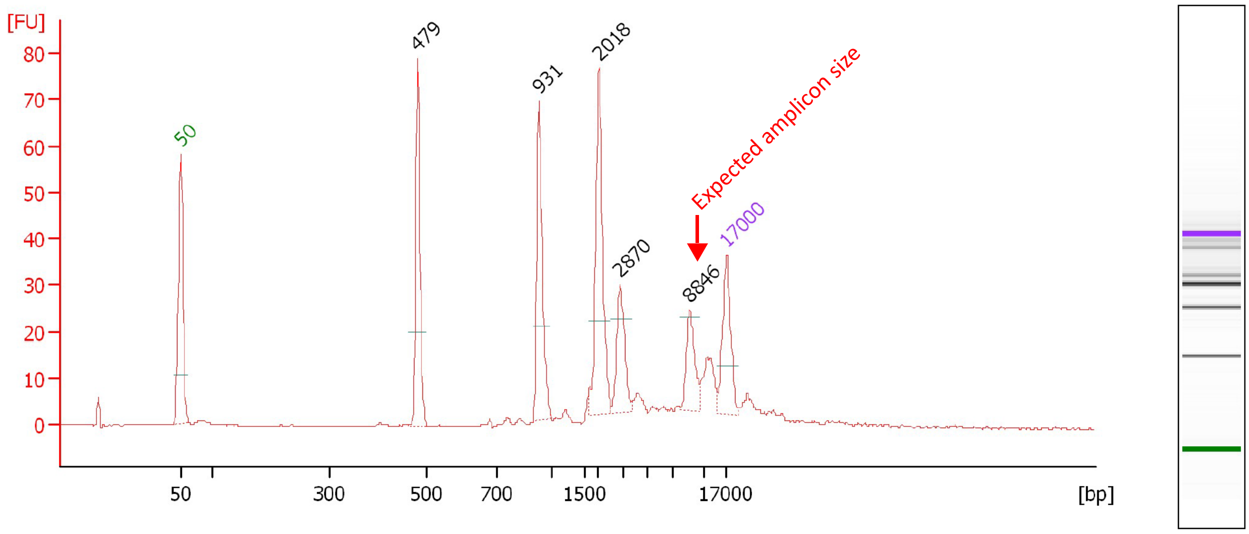

Figure S1b

The same amplicon after gel extraction (see Figure 1, main text)

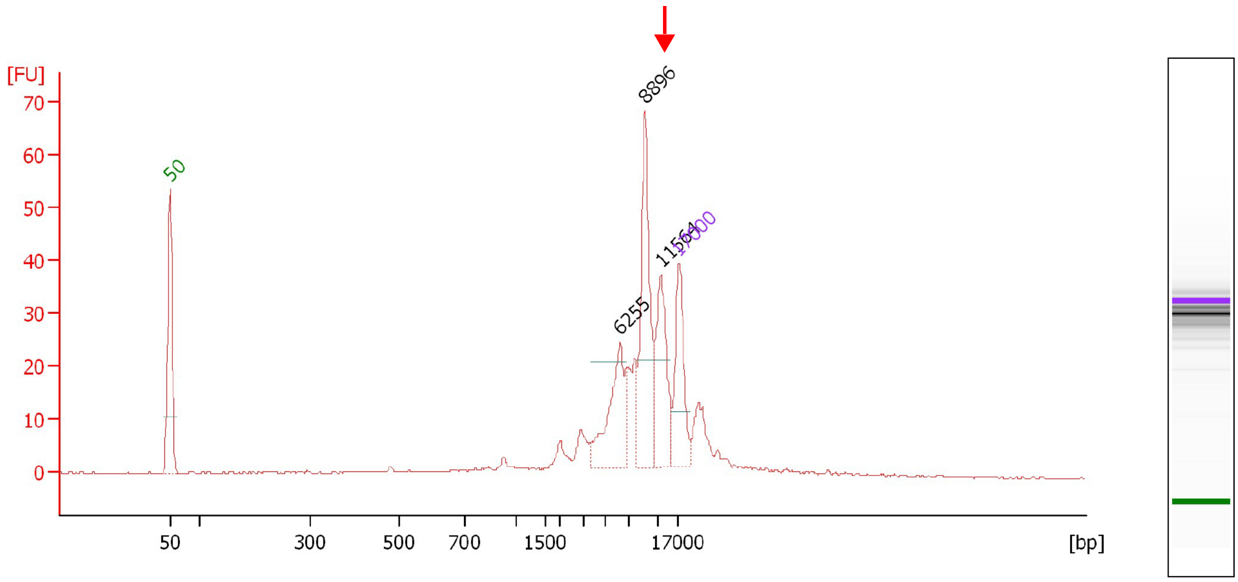

Supplement: Supplementary file 4 — Supporting Information Figure S1(PDF 367 kb) [file 41437_2018_70_MOESM4_ESM.pdf]
